# To Compare Time-Weighted Graphs to Evaluate the Inclination of the Acetabular Component of Patients Who Had Total Hip Replacement Surgery

**DOI:** 10.1155/2015/129610

**Published:** 2015-08-30

**Authors:** Leman Tomak, Yuksel Bek, Yılmaz Tomak

**Affiliations:** ^1^Department of Biostatistics and Medical Informatics, Faculty of Medicine, Ondokuz Mayis University, 55139 Samsun, Turkey; ^2^Department of Orthopedics and Traumatology, Faculty of Medicine, Ondokuz Mayis University, 55139 Samsun, Turkey

## Abstract

Time-weighted graphs are used to detect small shifts in statistical process control. The aim of this study is to evaluate the inclination of the acetabular component with CUmulative SUM (CUSUM) chart, Moving Average (MA) chart, and Exponentially Weighted Moving Average (EWMA) chart. The data were obtained directly from thirty patients who had undergone total hip replacement surgery at Ondokuz Mayis University, Faculty of Medicine. The inclination of the acetabular component of these people, after total hip replacement, was evaluated. CUSUM chart, Moving Average chart, and Exponentially Weighted Moving Average were used to evaluate the quality control process of acetabular component inclination. MINITAB Statistical Software 15.0 was used to generate these control charts. The assessment done with time-weighted charts revealed that the acetabular inclination angles were settled within control limits and the process was under control. It was determined that the change within the control limits had a random pattern. As a result of this study it has been obtained that time-weighted quality control charts which are used mostly in the field of industry can also be used in the field of medicine. It has provided us with a faster visual decision.

## 1. Introduction

Statistical process control (SPC) methods, utilizing the increasingly available routinely collected electronic patient records, could be used in continuous monitoring of clinical outcomes using routinely collected data [1, 2]. The “control charts” are a sequential analysis statistical tool that is particularly suited to the identification of small changes or changes in the number of cases, in one direction or another [1–3].

Control chart is an effective tool to identify specific causes of extreme variability. To distinguish the causes of variability, one of the most important visible signs is extreme observation on the control chart [1–3]. If some observations are outside the control limits, it means that the process is an out-of-control process, and the balance of the process is deteriorated. So corrective operations are needed so that the process can be controlled [2, 4]. If the process is in-control, the test results of patients are reported, but if it is out-control, the process is rejected and the results of tests are not reported [5].

The different control charts can be used for the evaluation process. While some of them are suitable for attribute data such as* p*-chart,* np*-chart,* c*-chart, and* u*-chart; some are used for continuous data such as individual, moving range, X-bar, range, standard deviation.

There are also other control charts which are used for various purposes apart from these. The most important of them are time-weighted control charts. They are preferred in some situations where Shewhart charts did not detect the case of small shifts. There are CUmulative SUM chart, Moving Average chart, and Exponentially Weighted Moving Average (Geometric Moving Average) chart in this group [1, 4, 6, 7].

In the last decade, there has been a growing interest in the application of these techniques to the medical practice, especially evaluating and often promoting the use of control charts assessment of performance in three main areas: trainees' acquisition of competence in procedural skills [8, 9]; quality control at departmental or organizational level [10]; and performance of specialists [11, 12]. In medicine, some control chart techniques are commonly utilized.

Total hip replacement (THR) is the one of the most commonly performed adult reconstructive orthopedic procedures. However, technological advances improve successes of THR, and most of the failures of THRs are related to technical faults. Proper placement of the acetabular and femoral components in the frontal and axial plane is very important for successful THR procedures. Abnormal acetabular component inclination causes early acetabular loosening and revision surgeries. Acetabular component inclination is very important technical criteria for languidly of THR [13].

Though control charts have been well researched and developed in the last decade, it is true that many qualified practitioners do not use them, even though there may be justifiable reasons to make use of this technique. Possibly this is due to lack of instruction on control charts in many classes on SPC.

We aimed to develop and test a CUmulative SUM (CUSUM) chart, Moving Average (MA) chart and Exponentially Weighted Moving Average (EWMA) chart for monitoring the inclination of the acetabular component after total hip replacement.

## 2. Materials and Methods

The data for the time-weighted control chartswere obtained by using X-rays that were obtained directly from thirty people who had had total hip replacement surgery at Ondokuz Mayis University, Faculty of Medicine.

There are several technical criteria such as acetabular component inclination, acetabular anteversion, femoral offset, and femoral stem alignment for determination of optimal THR procedures. Standardized anteroposterior (AP) postoperative radiographs were analyzed using goniometer to measure acetabular component inclination [13].

**Figure 1:**
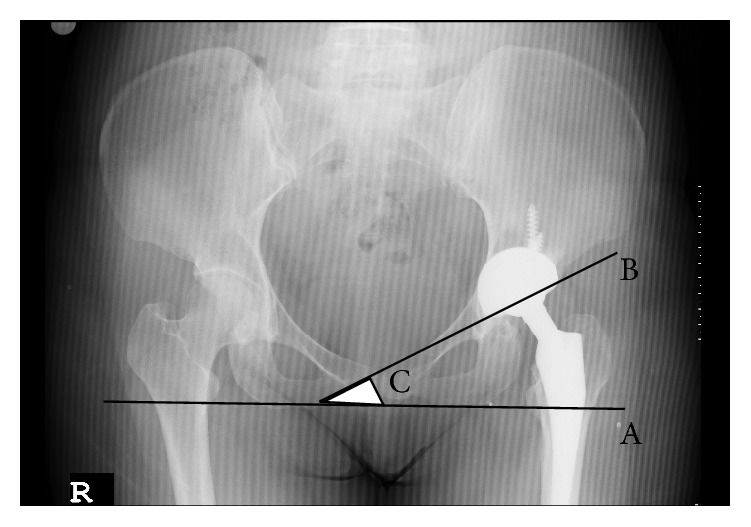
Measurement of the acetabular inclination angle. A. Line tangential to the ischial tuberosities. B. Line through the axis of the major diameter formed by apex of superior and inferior edges of the acetabular component on the radiograph. C. Acetabular inclination angle.

The inclination of the acetabular component was measured using the angle between a line joining the ischial tuberosities and a line crossing the long axis of the acetabular component, determined by means of the axis of the major diameter that is formed by the apex of superior and inferior edges of the acetabular component on the radiograph (Figure 1) [13].

The data for the acetabular inclination angles was obtained from total hip replacement patients' radiographs retrospectively. Although the target value is 45° for this angle, it is considered to be normal that the angle varies from 30 degrees to 50 degrees [13].

CUSUM chart, Moving Average chart, and Exponentially Weighted Moving Average are used to evaluate quality control process of the acetabular inclination angles [1, 4, 6, 7]. MINITAB Statistical Software 15.0 was used to get variable control charts [14].

### 2.1. CUmulative SUM Chart

Control material will be monitored by control methods for at least 20 days. At the end of this process, the values of mean and standard deviation of the results obtained are determined. A chart is generated using these statistics [4, 6, 15].

The values of CUmulative SUM (CUSUM) of deviations from the average are written on *Y*-axis. The measuring time or the number of observation is located on *x*-axis. Lower and upper confidence intervals are plotted as multiples of a certain standard deviation [4, 6, 15].

Given a sequence of values (*X*_*i*_) generated from a process, a CUSUM statistic is formed by plotting the quantity [1, 3]

(1)  Ci=∑j=1iXj−μ0 or

(2)  Ci=∑j=1iXj−μ0σ,

where *μ*_0_ is the process mean or target value, *σ* is the process standard deviation, *i* number of the sample groups, and *j* : 1,2,…, *i*. It is assumed that *σ* is known [1, 3]. When *σ* is unknown, *σ* is estimated as $\overset{-}{MR}/d_{2}$ or *S*/*c*_4_ [7].

Equation (2) is often known as the standardized CUSUM or scaled CUSUM. As long as the process is considered to be in-control, the average of *C*_*i*_ will be 0. It may be shown that the variance of *C*_*i*_ is *i* · *σ* ^2^ in (1).

Different procedures are used for CUSUM statistics, the most important of which are Tabulation CUSUM and the V-Mask CUSUM [1, 3, 6, 7, 16].

### 2.2. Tabulation CUSUM

CUSUM charts can be designed to detect a positive deviation or negative deviation from the average of process “*μ*_0_.” It is assumed that the standard deviation of process does not change [3].

*C* ^+^ which is designed to determine the increase in the average of process and *C* ^−^ which is designed to determine the reduction in the average of process are created primarily.

These are also called one-sided upper CUSUM and one-sided lower CUSUM [1, 6, 7, 17]:

(3) Ci+=max0,Xi−μ0+K+Ci−1+,Ci−=max0,μ0−K−Xi+Ci−1−.

Initial values are taken as *C*_0_^+^ = *C*_0_^−^ = 0.

*K* is called a reference value. If the shift is indicated in the form of *μ*_1_ = *μ*_0_ + *δ* · *σ*, the value of *K* is evaluated as half of the slip (the median between the two values) [1, 3]:

(4) $$\begin{array}{c} K=\frac{\delta }{2}\cdot \sigma =\frac{\left|\mu_{1}-\mu_{0}\right|}{2}. \end{array}$$

The value of *k* is the median value in the unit of one standard deviation [1, 3]:

(5) $$\begin{array}{c} k=\frac{K}{\sigma }=\frac{\left|\mu_{1}-\mu_{0}\right|}{2\sigma }. \end{array}$$

After the definition of the function, the control limit (*H*) is determined, and this value is often referred to as the “decision interval” [1, 3, 6]:

(6) $$\begin{array}{c} H=h\cdot \sigma . \end{array}$$

If either *C*_*i*_^+^ or *C*_*i*_^−^ is larger than *H*, the process is accepted to be out of control. The choice of the parameters “*h*” and “*k*” determines the performance of the CUSUM chart. Using *h* = 4 to 5 and *k* = 0.5 is generally preferred, because this provides a CUSUM chart that has good ARL properties against a shift of about 1*σ* in the process mean [1, 3].

When either *C*_*i*_^+^ or *C*_*i*_^−^ is larger than *H*, in order to bring the process back to the target value *μ*_0_ it may be helpful to have an estimate of the new process mean following the shift. The average of the new process is determined as shown below [1]:

(7) Ci+>H⟹μ^=μ0+K+Ci+N+,Ci−>H⟹μ^=μ0−K−Ci−N−.

Some researchers propose a technique of “standardized CUSUM.” To create this chart, the value of *X*_*i*_ must first be standardized [1, 3, 6]:

(8) $$\begin{array}{c} Y_{i}=\frac{X_{i}-\mu_{0}}{\sigma }. \end{array}$$

Standardized CUSUM values are obtained as follows [1, 3]:

(9) Ci+=max0,Yi−k+Ci−1+,Ci−=max0,−k−Yi+Ci−1−.

### 2.3. V-Mask CUSUM

A commonly used approach is to evaluate on the basis of the slope of the CUSUM line. This is V-Mask CUSUM.

“V” mask consists of a peak point (P), a localization point (O), and two slope lines acting as decision line (LDL and UDL lines). “V” mask consists of a peak point (P point), a localization point at the pullback distance from the peak point (O point), and 2 slope lines serving as decision lines (LDL and UDL lines). “*w*” value is used to demonstrate how many observations should be shifted to get the moving average [1, 4, 6, 16].

The vertex of the V mask is placed at a certain distance in front of the most recent observation (*C*_*i*_) on the control chart. If all values placed on the table remain inside the angle formed by lines of V-Mask, the process is considered to be in control. If any of the points is located outside the angle, process is considered to be out of control [4].

V-Mask CUSUM is applied to sequential values [1]:

(10) $$\begin{array}{c} C_{i}=\overset{i}{\underset{j=1}{\sum }}Y_{j}=Y_{i}+C_{i-1}. \end{array}$$

According to the above formula, “*Y*_*i*_” is standardized value of observation point [1]:

(11) $$\begin{array}{c} Y_{i}=\frac{\left(X_{i}-\mu_{0}\right)}{\sigma }. \end{array}$$

The performance of V mask is determined by the lead distance (*d*) and “*θ*” angle. If the following equations are accepted, tabular CUSUM and V-mask are equal [1]:

(12) $$\begin{array}{c} k=A\cdot \tan\theta ,\;h=A\cdot d\cdot \tan\theta =d\cdot k. \end{array}$$

For *A* = 1  (*k* = 1/2  and  *h* = 5) these equations are [1]

(13) $$\begin{array}{c} \frac{1}{2}=1\cdot \tan\theta ⟹\theta =26.57,\;5=d\cdot \frac{1}{2}⟹d=10. \end{array}$$

### 2.4. Moving Average Chart

During the creation of this chart, the individual observations are collected as *X*_1_, *X*_2_,…, *X*_*i*_. The moving average value is determined as follows at the “*w*” distance and the “*i*” time [1]:

(14) $$\begin{array}{c} M_{i}=\frac{X_{i}+X_{i-1}+\cdots +X_{i-w+1}}{w}. \end{array}$$

The “*w*” value is used to show how many observations should be slided to obtain moving average [7].

Variance of the moving average (*M*_*i*_) is as follows [1]:

(15) $$\begin{array}{c} V\left(M_{i}\right)=\frac{1}{w^{2}}\overset{i}{\underset{j=i-w+1}{\sum }}V\left(X_{j}\right)=\frac{1}{w^{2}}\overset{i}{\underset{j=i-w+1}{\sum }}\sigma^{2}=\frac{\sigma^{2}}{w}. \end{array}$$

Control limits for this chart are as follows [1]:

(16) UCL=X−+3·σ^w,CL=X−,UCL=X−−3·σ^w.

The value of $\overset{-}{MR}/d_{2}$ is used to estimate *σ*. Control limits are [7]

(17) UCL=X−+3·MR−d2·w,CL=X−,UCL=X−−3·MR−d2·w.

When the values of mean and standard deviation are given, the upper and lower limits would be as follows [1]:

(18) UCL=μ0+3·σ0w,CL=μ0,LCL=μ0+3·σ0w.

The control procedure consists of the calculation of the new moving average (*M*_*i*_) value for each observation and the marking of these on the chart. If one of the values is located outside the boundaries, the process is considered to be out of control [1].

### 2.5. Exponentially Weighted Moving Average Chart

The EWMA statistic is defined for the observations of *X*_*i*_ with mean *μ* and variance *σ* ^2^ as follows [1, 3, 6, 7, 16, 18]:

(19) $$\begin{array}{c} Z_{i}=\left(1-\lambda \right)\cdot Z_{i-1}+\lambda \cdot X_{i}, \end{array}$$

where *i* = 1,2,…, *λ* is a parameter (0 < *λ* ≤ 1). The starting value *Z*_0_ is equal to the process mean [1, 3, 6]. *Z*_*i*_ is a weighted average of the *X*_*j*_  (*j* = 1,2,…, *i*) and *μ* with the weights of *X*_*j*_ decreasing exponentially [1, 3]:

(20) $$\begin{array}{c} Z_{i}=\lambda \overset{i-1}{\underset{j=0}{\sum }}\left[{\left(1-\lambda \right)}^{j}X_{i-j}\right]+{\left(1-\lambda \right)}^{i}Z_{0}. \end{array}$$

The weight of the current observation is *λ*, and that of the previous one is *λ*(1 − *λ*). The older the observation is, the smaller the value will be *λ*(1 − *λ*)^*j*^ with which it is weighted. The weight of the observation being studied at the moment will be *λ* and the weight of the previous observation will be (1 − *λ*), and it will gradually decrease [3]. The sample average of the *λ*(1 − *λ*)^*j*^ weights decreases geometrically by time. When the sums of the weights are combined, the formula may be shown as follows [1]:

(21) $$\begin{array}{c} \lambda \overset{i-1}{\underset{j=0}{\sum }}{\left(1-\lambda \right)}^{j}=\lambda \cdot \left[\frac{1-{\left(1-\lambda \right)}^{i}}{1-\left(1-\lambda \right)}\right]=1-{\left(1-\lambda \right)}^{i}. \end{array}$$

The variance of *Z*_*i*_ is [1, 3]

(22) $$\begin{array}{c} \sigma_{Z_{i}}^{2}=\left(\frac{\lambda }{2-\lambda }\right)\left[1-{\left(1-\lambda \right)}^{2i}\right]\sigma^{2}. \end{array}$$

When *i* is large, the variance can be shown as follows [3]:

(23) $$\begin{array}{c} \sigma_{Z_{i}}^{2}=\left(\frac{\lambda }{2-\lambda }\right)\sigma^{2}. \end{array}$$

Control limits are [1, 3]

(24) UCL=X−+k·σ·λ2−λ1−1−λ2i,CL=X−,LCL=X−−k·σ·λ2−λ1−1−λ2i.

When *i* > 10, the formula for the control limits can be a simplified formula, and “*k*” value is considered to be typically 3 [1, 3, 6, 7, 16, 17]:

(25) UCL=X−+3·σ^·λ2−λ,CL=X−,LCL=X−−3·σ^·λ2−λ.

If *σ* is not known, *σ* is estimated by using either ${\overset{-}{MR}/d}_{2}$ or *S*/*c*_4_ [19]. If *μ* and *σ* are known, the control limits are as follows [1, 3]:

(26) UCL=μ0+3·σ·λ2−λ,CL=μ0,LCL=μ0−3·σ·λ2−λ.

## 3. Results

**Figure 2:**
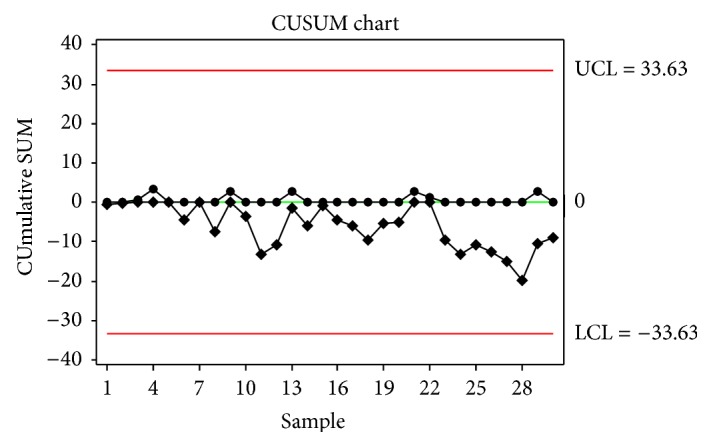
CUSUM Chart.

Time-weighted control charts were obtained using the acetabular inclination angles of thirty patients. CUSUM chart is shown in Figure 2. It is obvious that all the observations are within the control limits and the process is stable.

**Figure 3:**
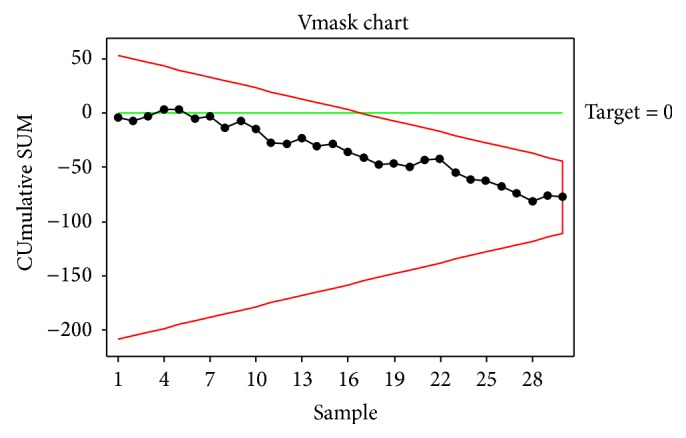
V-Mask CUSUM Chart.

All the observations are within both UCL and LCL on V-Mask CUSUM chart (Figure 3). The process is under control.

**Figure 4:**
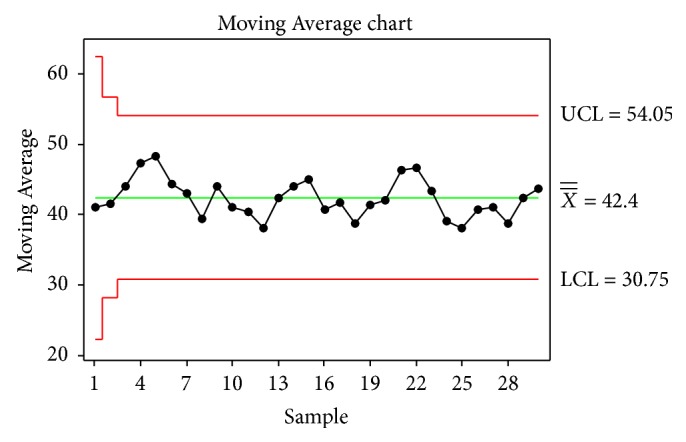
Moving Average Chart.

Moving Average chart is shown in Figure 4. Since all of the observations are settled within control limits, it is clear that the process is stable.

**Figure 5:**
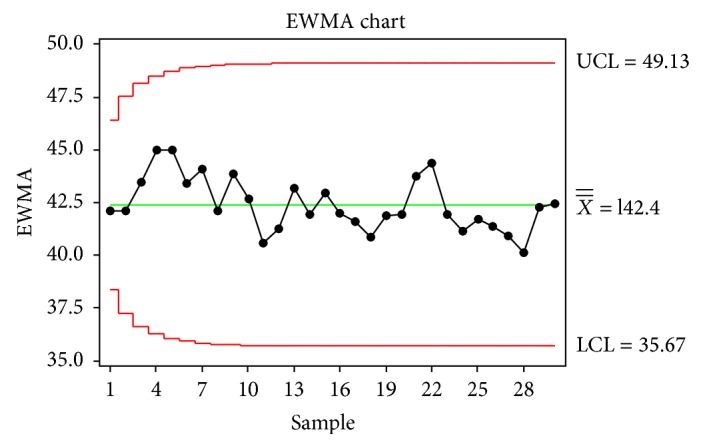
EWMA Chart.

EWMA chart is given in Figure 5. There is no observation settled outside of the control bounds. Because of this random pattern, the process is stable.

## 4. Discussion

Time-weighted charts are used as an alternative to Shewhart charts in detecting small changes. Although Shewhart charts are effective in detecting changes greater than 2*σ*, they are not effective enough to detect the changes between 0.5 and 1.5*σ*. So, time-weighted charts are preferred to detect such changes [6].

CUSUM chart is a precise and quantitative chart which is used to demonstrate if this difference is random or systematic [1, 4, 6, 7].

In this study, CUSUM, EVMA, and MA charts were used to evaluate the inclination of the acetabular component in patients who underwent hip replacement surgery. The evaluation done with time-weighted charts revealed that the acetabular inclination angles were within the control limits and the process was stable. It was demonstrated that the change within the control limits had a random pattern.

CUSUM statistics occur in a random pattern around zero of the variation. Random scatter around zero axis is the expected pattern, but if cumulative values display a continuous increase or decrease towards a particular direction at a certain point, then the observation values are inspected from this point on. A steep slope (gradient) indicates that there is a systematic error and that the observations are out of control [1, 4, 6].

There was no steep slope on the drawn charts for the inclination of the acetabular component in patients who underwent hip replacement surgery.

One of the advantages of charted CUSUM is the horizontal lines it has for the control limits. This allows charted CUSUM to be interpreted as Levey-Jennings chart. This type of interpretation requires less experience and evaluation. Thus, when a large number of analysts use the control process, the interpretation will be more consistent. Another advantage of this method is that it is applicable with only scaled procedures (without drawing any tables).

However, when compared with Levey-Jennings control chart limited with 3 s, CUSUM procedure is better in detecting systematical errors but less sensitive in detecting random errors. Hence, rather than being used alone, it should be used either together with Levey-Jennings procedure or as a separate table with the Levey-Jennings table. Although interpretation is easier and more objective with this technique, determining the threshold and control limits is somewhat difficult.

V-mask CUSUM technique is highly objective in interpreting data; however, it is not widely used in clinical laboratories or recommended. Instead, interpretation depends on visual inspection and evaluation of the angle of CUSUM line.

Sometimes, a special graph paper with 45-degree angle shapes is also used. The aim in using this specific graph paper is to scale the graph so that a 2 s change on *y* axis will be the same as the distance between the 2 points on *x* axis. Thus, when the observed mean is about 2 s from the expected mean, the 45-degree angle represents the expected slope/gradient [4].

Moving Average chart and EWMA chart are used in cases where Shewhart control charts are not able to detect small changes. The performance of EWMA control chart is almost equal to that of CUSUM chart, and it is an easily available and applicable chart. CUSUM, MA, and EWMA charts are ideal for the inspection of the individual observations.

The CUSUM technique has several methodological characteristics that make it perform better than the commonly used healthcare quality control tools such as incident reporting and clinical audit or indeed any other methods that report aggregate results. This graphical technique is associated with false positive and false negative states. The choice and placement of chart limits determine which of the two states is more likely to occur, and reducing the likelihood of one increases that of the other. CUSUM methods can be used in continuous monitoring of clinical outcomes of the inclination of the acetabular component in patients who underwent hip replacement surgery. Used prospectively, they could lead to the prompt detection of periods of suboptimal standards of the hip replacement surgery.

With this study, it has been demonstrated that time-weighted quality control charts, which are used mostly in the field of industry, can also be used in the field of medicine. It has also been demonstrated that they allow a faster visual decision.
